# Macrolide-resistant *Mycoplasma genitalium* infections in Cuban patients: an underestimated health problem

**DOI:** 10.1186/s12879-018-3523-9

**Published:** 2018-11-29

**Authors:** Brian Arturo Mondeja, Javier Couri, Nadia Maria Rodríguez, Orestes Blanco, Carmen Fernández, Jørgen Skov Jensen

**Affiliations:** 1 Pedro Kourí Tropical Medicine Institute, La Habana, Cuba; 2 Faculty of Biology, University of Havana, La Habana, Cuba; 3 Statens Serum Institut, Research Unit for Reproductive Tract Microbiology, Artillerivej 5, DK-2300 Copenhagen S, Denmark

**Keywords:** *Mycoplasma genitalium*, Antimicrobial susceptibility, Macrolide resistance, Sexually transmitted infections

## Abstract

**Background:**

The increasing prevalence of macrolide resistant *Mycoplasma genitalium* is a major concern worldwide. In Cuba, several cases of clinical treatment failure with 1 g single dose and extended azithromycin regimen have been detected and the aim of the present investigation was to retrospectively determine the prevalence of macrolide-resistance mediating mutations (MRMM) in *M. genitalium*-positive samples conserved at the Cuban National Reference Laboratory of Mycoplasma Research between 2009 and 2016.

**Methods:**

A total of 280 positive DNA extracts were analysed by a 5’ nuclease assay for detection of *M. genitalium* MRMM. Ten urogenital specimens from patients with azithromycin treatment failure and MRMM were inoculated in Vero cell to obtain the isolates for subsequent determination of antimicrobial susceptibility.

**Results:**

The overall prevalence of MRMM was 32%. No MRMM was detected in samples collected between 2009 and 2013 but since 2014 a dramatic increase to 90% (95% CI, 76–96%) in 2016 was seen. Three new *M. genitalium* isolates were isolated in Vero cell cultures and confirmed phenotypic resistance to macrolides in a cell-culture assisted susceptibility test. Preliminary observations suggest that combination therapy with levofloxacin and doxycycline may represent an affordable option for treatment of macrolide resistant *M. genitalium* infections.

**Conclusions:**

This investigation showed the rapid emergence and high prevalence of MRMM in *M. genitalium*-infected patients in Cuba and confirmed the phenotypic resistance in isolates carrying MRMM. We suggest that Cuban guidelines for sexually transmitted infections are modified to include testing for *M. genitalium* and detection of MRMM in patients with failure of syndromic treatment, to ensure that in these cases, the treatment will be guided by etiologic diagnosis.

## Background

*Mycoplasma genitalium* is an emerging sexually transmitted pathogen and the increase of macrolide resistance is considered a health problem globally [1]. In this bacterium, macrolide resistance is mediated mainly by point mutations in the A2058 and A2059 (*Escherichia coli* numbering) positions in region V of the 23S ribosomal RNA. These mutations are associated with azithromycin treatment failure and high minimal inhibitory concentration (MIC) for macrolides as documented in several *M. genitalium* strains [2, 3].

The prevalence of strains with macrolide resistance mediating mutations (MRMM) is highly variable and unknown in many regions. In Europe, Australia and Asia, it is generally above 30% and in extreme cases, as in Greenland, 100% of *M. genitalium* strains carry MRMM [4–8]. In Cuba, macrolides are used as the first line treatment for urogenital syndromes caused by sexually transmitted infections (STI), including *M. genitalium* and chlamydia infections. Since 2007, *M. genitalium* diagnosis has been performed at the Pedro Kourí Tropical Medicine Institute (IPK), and several cases of treatment failure with 1 g single dose and extended azithromycin regimens have been detected in the IPK - STI clinic within recent years. In 2015, a new macrolide resistant *M. genitalium* strain (B19, A2059G mutation) was isolated from one of these patients using Vero cell co-culture [9], and antimicrobial susceptibility patterns were determined using a cell-assisted procedure [10]. However, no data about the prevalence of MRMM carrying *M. genitalium* strains in Cuba are available, but clinical experience of macrolide treatment failures and the isolation of at least one MRMM strain suggest the possible circulation of MRMM carrying *M. genitalium* strains in Cuban patients after 2015, which has direct implications for the effectiveness of syndromic management of STI.

The aim of the present investigation was to retrospectively determine MRMM prevalence in *M. genitalium*-positive samples conserved at the Cuban National Reference Laboratory of Mycoplasma Research on IPK between 2009 and 2016.

## Materials and methods

### Study design

A retrospective study was conducted at the Cuban National Reference Laboratory of Mycoplasma Research – IPK, for the direct detection of MRMM in *M. genitalium* positive clinical samples archived since 2009. This study was approved by The Pedro Kourí Tropical Medicine Institute Ethical Board (approval CEI-IPK 57–16) and written informed consent to participate and publish was obtained from all patients.

### *M. genitalium* positives specimens

For the study, a total of 280 *M. genitalium* positive DNA extracts from Cuban patients with urogenital syndromes, spontaneous abortion and infertility were analysed. These were representing all samples submitted to IPK for *M. genitalium* diagnosis since 2009 and up to December 2016. DNA was extracted by the Chelex 100 method and conserved at − 80 °C [11]. Until December of 2014, *M. genitalium* diagnosis was performed by a 16S rRNA singleplex-PCR with internal control [12] and confirmation by a qPCR based in the amplification of *mgpB* gene [11]). Since January of 2015, *M. genitalium* diagnosis was performed by the same *mgpB* gene qPCR mentioned above and the confirmation was with a *mgpA* gene – qPCR [13]. For each patient, only the first positive specimen was included in the study.

### Detection of MRMM

DNA specimens were analyzed by a modification of the 5′ nuclease genotyping assay for *M. genitalium* MRMM testing, described by Kristiansen et al. [14]. In brief, a qPCR mix was prepared in a final volume of 25 μL containing 500 nM of the 23S rRNA gene primers described by Jensen et al. [15], 200 nM of wild-type probe: Cy5-GGA CGG AAA GAC CCC GTG AAG CTT T-BHQ2, 100 nM of each MRMM probes MRMM-A2058G: FAM-GAC GGG AAG ACC CCG TGA AGC TTT-BHQ1 and MRMM-A2059G: FAM- GAC GGA GAG ACC CCG TGA AGC TTT-BHQ1 [14], and 1.5 U of Top*Taq*-polymerase (Qiagen,Hilden, Germany). As positive controls for MRMM, DNA from *M. genitalium* strains M6271 (A2058G), M6489 (A2059G) and M6302 (A2058C) were used, and as wild-type *M. genitalium* strains the Danish strains M2300, M2321 and M2341 were used [3].

Samples were amplified and analysed in a Rotor-Gene Q 5-plex instrument (Qiagen) using the same amplification program as described by Kristiansen et al. [14]. Results were analysed using the Rotor-Gene Q Software Version 2.1.0.9 (Qiagen) by the Scatter Analysis module with the reading combination of the green and red fluorescence channel.

### Isolation and determination of antimicrobial susceptibility of MRMM strains from clinical specimens

Ten urogenital specimens from patients with azithromycin treatment failure and with an *M. genitalium* load > 10,000 genome equivalents (geq) per mL, were inoculated in Vero cell as previously described [16] in an attempt to obtain new isolates. The specimens comprised seven male urethral swabs (three male homosexual couples and one heterosexual man) and three endocervical swabs (one of them from the female partner of the male patient providing the sample yielding the B19 strain previously described [10]). In order to document lack of cross-contamination between cell cultures, an *mgpB* based DNA typing method [17] was applied to primary samples and isolated strains.

Antimicrobial susceptibility testing was performed by a cell-assisted procedure as previously described, but with an incubation time of 28 days [10]. MICs to azithromycin, erythromycin, doxycycline, tetracycline, ciprofloxacin, ofloxacin, moxifloxacin and levofloxacin was defined as the lowest concentration of antimicrobial capable of inhibiting 99% of the *M. genitalium* growth in the test well compared with the *M. genitalium* growth in the control wells [18]. MRMM identification of the isolates was done by the nuclease genotyping assay described above, using genomic DNA of the isolate and of the corresponding clinical specimen.

### Treatment of patients with macrolide resistant *M. genitalium* infection

Between July 2015 and December 2017, all 46 *M. genitalium* positive patients with treatment failure after azithromycin (500 mg day 1 followed by 250 mg days 2–5) and attending the STD clinic of the IPK Hospital have been treated with doxycycline 100 mg 2 times daily plus levofloxacin 500 mg 2 times daily for 14 days. This regimen has been used due to the limited availability of moxifloxacin in Cuba. Microbiological cure was evaluated 30 days after treatment using the same PCR methodology as used for the primary diagnosis.

### Statistical analysis

The statistical comparisons between the frequency of resistant *M. genitalium* in each year of the study and for the proportion of MRMM between men and women was compared with Fisher’s exact test with p for trend (two-sided *p* values). Differences with *p* < 0.05 were considered statistically significant (StatsDirect version 3.1.14 (StatsDirect Ltd., Cheshire, UK)).

## Results

### Distribution of *M. genitalium* positive patients

**Fig. 1:**
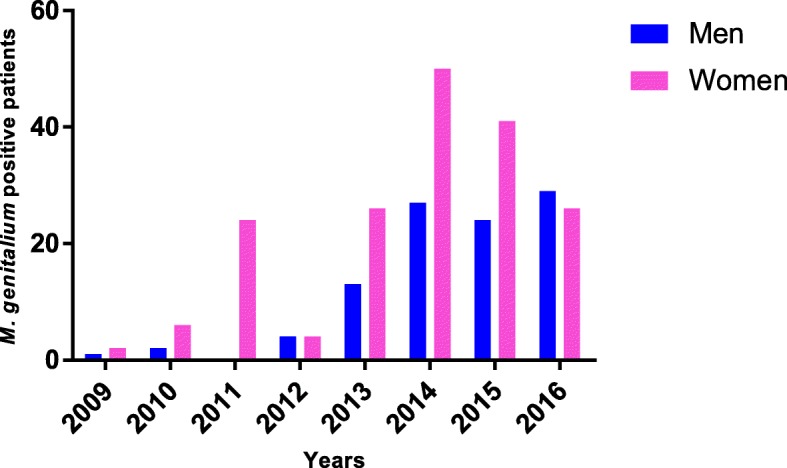
Gender distribution and number of *M. genitalium* positive patients per year of study

Of the 280 *M. genitalium* positive patients, 100 (35.7%) were men and 180 (64.3%) were women (Fig. 1).

### Prevalence of MRMM in the clinical specimens

**Fig. 2:**
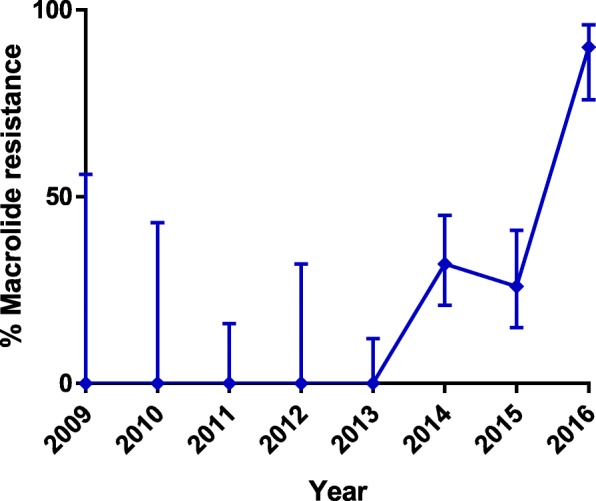
Prevalence of MRMM genotype in percent of clinical specimens per year of study. Error-bars show 95% confidence intervals

Of the 280 samples analysed, 202 (72%) were amplifiable by the MRMM qPCR. Of these 202, 138 (68%; 95% CI 57–81%) were identified as wild-type genotype (macrolide susceptible) and 64 (32%; 95% CI 24–40%) were identified as carrier of mutant genotypes. MRMM was not detected in any of the 65 successfully amplified samples collected between 2009 and 2013 0% (95% CI: 0–6%). In contrast, MRMM was detected in 64 of 137 clinical samples 47% (95% CI: 39–55%) collected since 2014 with a dramatic increase to 90% (95% CI: 76–96%) in 2016 (*p* < 0.0001, *p* < 0.0001 for trend) (Fig. 2).

No differences between the MRMM prevalence in men and women were detected (*p* = 0.88).

### Genotype distribution among MRMM positive samples

Out of a total of 64 MRMM positive samples, 52 samples (81%) were identified as A2058G/A2059G and 12 (19%) as A2058C/T. The latter genotype was more commonly found in female than in male specimens.

### Antimicrobial susceptibility pattern of MRMM strains from clinical specimens

Of the ten *M. genitalium* positive specimens, only three were capable of growing in the Vero cell culture and yielded new isolates. The B25 and B26 strains were isolated from a male couple and showed the same *mgpB* type (not previously described and denominated Cuba3; Acc# MH407699). The B30 strain was isolated from the female partner of the male patient yielding the B19 isolate previously reported [10]. The B30 strain showed an identical *mgpB* type to that of the B19 strain (genotype 4). For all three isolates, the same *mgpB* type was detected from isolate and the corresponding clinical specimen.

The three new clinical *M. genitalium* isolates were resistant to macrolides with a MIC value of > 8 mg/L. MIC values for tetracyclines were 0.5–2 mg/L and for quinolones, a MIC of 0.125 mg/L was found for moxifloxacin (Table 1).

**Table 1:** Minimal Inhibitory Concentrations (MICs) for the three MRMM carrying Cuban *M. genitalium* isolates as determined by a modified cell-culture-based method

*M. genitalium* isolates		MIC VALUES (mg/L)							
	MRMM	Azithromycin	Erythromycin	Ciprofloxacin	Ofloxacin	Levofloxacin	Moxifloxacin	Tetracycline	Doxycycline
B25	A2059G	> 8	> 8	4	1	2	0.125	1	0.5
B26	A2059G	> 8	> 8	4	1	0.5	0.125	1	0.5
B30	A2059G	> 8	> 8	8	1	1	0.125	2	0.5

All the isolates and the corresponding clinical specimen were identified as MRMM A2058G/A2059G by the 5' nuclease assay and as A2059G by sequencing (Table 1) clearly confirming the good concordance between detection of MRMM by the molecular assay and the demonstration of a phenotypic resistance in vitro in the cell-assisted assay as well as in vivo documented by macrolide treatment failure.

### Treatment of patients with macrolide resistant *M. genitalium* infection

All 46 cases with azithromycin treatment failure were found to carry *M. genitalium* with MRMM. Dual therapy with doxycycline and levofloxacin for 14 days eradicated *M. genitalium* from all patients as documented by PCR 30 days after treatment.

## Discussion

The emergence of MRMM in *M. genitalium* in several countries is a worrying reality. For several years, this sexually transmitted pathogen has been underestimated as responsible for STI syndromes in men and women. In the Unites States Center for Diseases Control STI treatment guidelines the infection got attention with a separate chapter for the first time in 2015 [19]. In 2016 the European guideline on *Mycoplasma genitalium* infections was published as the first international guideline addressing diagnosis and treatment [20]. This was the first step to harmonize the international recognition and treatment of *M. genitalium* as an STI.

Since 2004, *M. genitalium* infections have been studied at IPK, and diagnosis was established in 2009 as part of an aetiological surveillance of STIs in Cuba. For many years, empiric treatment with macrolides and tetracyclines was used for the control of mycoplasma and chlamydia infections. In 2001, Cuba implemented syndromic management of STI, and the 1 g single dose azithromycin treatment became the recommended treatment for non-gonococcal urogenital infections in men and women. At this time, few studies were available that evaluated the treatment efficacy of macrolides and the emergence of resistance was an unknown problem. The latter, has become recognised as a worldwide problem after 2008 [2].

In the present investigation, we have determined the presence of MRMM in *M. genitalium* in Cuban clinical specimens archived since 2009, nine years after the initiation of azithromycin syndromic treatment of urogenital syndromes, and until 2016. From our data, it appears that *M. genitalium* with MRMM was very rare in Cuba before 2014. However, the number of analysed *M. genitalium* positive samples between 2009 and 2013 was relatively low, so it is difficult to make definite conclusions. Nevertheless, the explosion in the prevalence of *M. genitalium* with MRMM reaching 90% in 2016 is extremely worrying and threatening the current recommended syndromic management. In Cuba, more than 4000 cases of urogenital syndromes (lower abdominal pain, vaginal discharge and urethral syndrome) are reported to the Ministry of Public Health every year. However, it is not common that *M. genitalium* tests are ordered, even when macrolide treatment has failed. This finding is very similar in other countries and is partly due to lack of awareness among clinicians, but also due to lack of testing availability in many settings.

The genotyping assay used for MRMM detection was able to characterise more than 70% of the *M. genitalium* positive specimens, compared with 94.5% reported in the original paper by Kristiansen et al. [14]. However, when we analysed samples from 2016, 15 of the 17 non-amplified specimens were successfully re-amplified by the *mgpB* qPCR. These samples showed an *M. genitalium* load < 1000 geq/mL which may explain the failure of amplification in the genotyping assay. Other factors such as destruction of DNA, even when conserved at -80 °C, could be playing a role in the success of the genotyping assay, as has been shown for another *M. genitalium* molecular test [21]. Unfortunately, it was not possible to provide information about the exact positions of the MRMM as the method applied did not provide this level of discrimination. Most of the samples were used up for culture attempts and additional PCRs for MRMM was not possible. In Cuba, Sanger sequencing is difficult and expensive and consequently, a qPCR for the detection of MRMM directly from the clinical samples was introduced. Furthermore, as the type of mutation is not relevant for the clinical management, and as an increasing amount of surveillance data is produced by PCR based testing, even in more resourced settings, we do not consider this to be a major limitation of the study.

The three new Cuban *M. genitalium* strains isolated from samples with MRMM have an antimicrobial resistance profile with susceptibility to tetracyclines and moxifloxacin according to CLSI guidelines for *M. pneumoniae* [22]. The patient from whom the B25 strain was isolated is a Cuban man living in Switzerland, where he was infected, diagnosed and treated for *M. genitalium*. His Cuban male partner never lived outside of Cuba and was infected after unprotected sex during a visit of patient B25. Thus, introduction of the MRMM *M. genitalium* strain from Europe is the most plausible explanation, and confirmation of the same *mgpB* type (Cuba3) in clinical samples and MRMM strains, support this. Fortunately, both the B25 and B26 strains were susceptible to doxycycline and the patients were cured after combined treatment with doxycycline and levofloxacin for 14 days. The B30 female patient was a woman having had sex with foreign men and she was the sexual contact reported by the B19 male patient described in our previous study [10] at the time of *M. genitalium* diagnosis. The same *mgpB* type (type 4) was found in the clinical specimens and isolated strains B19 and B30, reaffirming the sexual transmission between the partners. However, it is impossible to determine if the MRMM strain was imported or not. Both patients were cured with levofloxacin plus doxycycline.

All three new isolates showed phenotypic resistance to macrolides. In these cases, a total concordance between the 5’nuclease genotyping assay, the macrolide resistance pattern determined by the Vero cell culture assay, and clinical and microbiological failure of azithromycin treatment was documented. This fact reaffirm the utility of the genotyping assay to predict azithromycin treatment failure in patients infected with *M. genitalium* when MRMM are detected. Unfortunately, no isolates with MRMM were obtained from the seven other clinical specimens inoculated in Vero cells.

In this investigation, the overall macrolide resistance rate was 32% but with all of the resistant strains detected within the last three years of the study. The A2058G/A2059G was the most frequently detected MRMM genotype detected in *M. genitalium* positives specimens as in most other studies. Comparable rates of macrolide resistance have been reported recently from Denmark [4], United Kingdom [6], and Australia [8], where the prevalence of macrolide resistance–associated mutations was 40, 41 and 43%, respectively. These studies also found similar distributions of the most common A2058G and A2059G mutations but did not see the same dramatic increase in the prevalence of MRMM strains. The A2058C/T was detected in 19% of Cuban MRMM specimens. Unfortunately, neither of the isolated strains had this mutation pattern but no influence on the MICs values for macrolides have been recorded in other investigations for the different mutation-types [2, 3].

Based on the findings reported here, a new molecular diagnostic strategy for *M. genitalium* at IPK has been established since January 2017. In this strategy, we use the *mgp*B qPCR as screening test and positive specimens are immediately confirmed by the 5’nuclease genotyping assay and by the *mgpA* qPCR for *mgpB* positive samples with less than 1000 geq/mL. This approach is in accordance with the European guideline on *M. genitalium* infections [20] where determination of MRMM is recommended for all *M. genitalium* positive samples.

The prevalence of MRMM carrying *M. genitalium* strains has been high after 2014 in Cuba. Since 2015, all *M. genitalium* positive patients with MRMM seen at the STD clinic at the IPK Hospital have demonstrated treatment failure after azithromycin. In these 46 cases, MRMM *M. genitalium* was successfully eradicated with a 14-days second-line combined therapy of levofloxacin plus doxycycline (Orestes Blanco, unpublished). This regimen has been used due to the limited availability of moxifloxacin in Cuba and may be applicable elsewhere. The choice of this combination regimen was based on the synergistic in vitro activity of doxycycline and moxifloxacin in fluoroquinolone susceptible *M. genitalium* strains [23]. Experimental work documenting the synergy between levofloxacin and doxycycline is currently underway. The high efficacy was somewhat unexpected as doxycycline monotherapy has a microbiological cure rate of approximately 30% and that of levofloxacin monotherapy is rarely exceeding 50% [24].

No data about MRMM prevalence in other Latin-American countries are available, but it will probably be found at a similar rate in countries with similar macrolide treatment strategy for STIs as Cuba.

## Conclusions

The results obtained in this investigation showed for the first time the rapid emergence and high prevalence of MRMM carrying *M. genitalium* strains in Cuban patients. No data about macrolide failure in syndromic STI management outside IPK are available, but macrolide resistance in *M. genitalium*-infected Cuban patients could be an underestimated health problem. We suggest that Cuban guidelines for STI treatment are modified to include testing for *M. genitalium* and detection of macrolide resistance–mediating mutations at least in patients failing syndromic treatment to ensure that in these cases the treatment will be guided by the aetiological diagnosis.

## Abbreviations

IPK: Pedro Kourí Tropical Medicine Institute
MRMM: Macrolide resistance mediating mutations
qPCR: Quantitative Polymerase Chain Reaction
STI: Sexually transmitted infections
